# Supplementary material for: miR-671-5p Inhibition by MSI1 Promotes Glioblastoma Tumorigenesis via Radioresistance, Tumor Motility and Cancer Stem-like Cell Properties
Source: Biomedicines. 2021 Dec 23;10(1):21. doi: 10.3390/biomedicines10010021 (PMC8773172; doi:10.3390/biomedicines10010021)
Supplement: Supplementary file 1 [file biomedicines-10-00021-s001.zip › biomedicines-1495456-supplementary.pdf]

Supplemental Table

Table S1. Primer used for qPCR.

Name	Forward	Reverse	Application
MSI1	AATTCCGTGTAGAGCGGACC	GCAGTGAGAGGAATGGCTGT	qRT-PCR
TRAF2	CGACCGTTGGGGCTTTGT	CCCCAGGAGGGTCTTGGAGAA	qRT-PCR
STAT3	GACATTCCCAAGGAGGAGGC	GGTCTTCAGGTATGGGGCAG	qRT-PCR
COL8A2	TGTGAACTGGGGTCATTCGG	CAGCCAGTCAGAAGTCAGCA	qRT-PCR
CDH11	GCCCTGTGACATTCCTTCGT	TTGAGCTCATCACGTCAGGG	qRT-PCR
IGFBP4	GACCCACTCCCAAAGCTCAG	CAACCAAGCAGATGGTGCAA	qRT-PCR
SGCG	ATTCTGTCTGTGGTAGAGCTCGG	TACTGCTCACGCACCATCTTAG	qRT-PCR
THBS1	GTCATAGCAACATTCACAGTTTGT	TCCACAAACGCAGCAAATTC	qRT-PCR
MMP2	CATCCAGACTTCCTCAGGCGG	GGTCCTGGCAATCCCTTTGTATG	qRT-PCR
PLOD1	AGCAGCATCCCTCGTTTCTG	AGAACTGAGCTGAGCGCTTG	qRT-PCR
PDLIM4	AGGCCATCAATGGTGAGAGC	ACAGTGTGAGGTGATCGTGG	qRT-PCR
EPCAM	TGGGTGAGATGCATAGGGAAC	AAGATGTCTTCGTCCCACGC	qRT-PCR
GCH1	TTGCGTACCTTCCTCAGGTG	TTCGAGGTCTGCGGCTAAAC	qRT-PCR
TFRC	AGAGCGTCGGGATATCGGGT	ATGAAGGGAGGACACGAGGG	qRT-PCR
PPAT	GTTGGTGCTTACACCTTGGC	ACATTCCTCTCGGATCCCCA	qRT-PCR
DDX21	TCCACGCGGTTGAGAAGAC	CCAGCGTCACTACGGAGTTT	qRT-PCR
CTSV	AGACCGCGGACGTCTGTAAT	TTTTGGAACAGCGGAGGCTAT	qRT-PCR
GAPDH	CTCATGACCACAGTCCATGC	TTCAGCTCTGGGATGACCTT	qRT-PCR

Supplementary Materials and Methods

Tunnel assay. After irradiation, GBM cells were harvested with mild trypsinization and then seeded into a six-well plate. We used the In Situ Cell Death Detection Kit (Roche) to stain the impaired DNA. The staining procedure was according to the manufacturer's instructions. We used microscopy to observe the dead cells. Alternatively, we harvested with mild trypsinization and washed it with PBS and applied it into flow cytometry. We quantified the amount with flow cytometry.

Transwell assay. The 24-well Insert System with an 8-μm pore-size polyethylene terephthalate membrane (Corning, NY) was used for the Transwell assay. The GBM cells were seeded in each chamber with a density of

2.5 × 10^4 cells in each well. The medium was removed after 24-hour incubation, and chambers were fixed by 100% methanol at room temperature for 30 mins. We stained the membrane with 50 μg/ml propidium iodide (Sigma Aldrich, MI) for 30 mins and counted with a fluorescent microscope.

Taqman microRNA analysis. MicroRNA was isolated from paraffin-embedded tissue sections from rectal cancer patients using Recover All™ Total Nucleic Acid Isolation Kit for FFPE (Life Technologies, Stockholm, Sweden). The microRNA quality was assessed by studying amplification efficiencies of microRNAs after serial dilutions of cDNA and measuring C.T. values of microRNAs using real-time PCR. miR-671-5p expression of GBM cells and patients was determined using Taqman microRNA assay and normalized to RNU6b.

Supplementary Figure S1

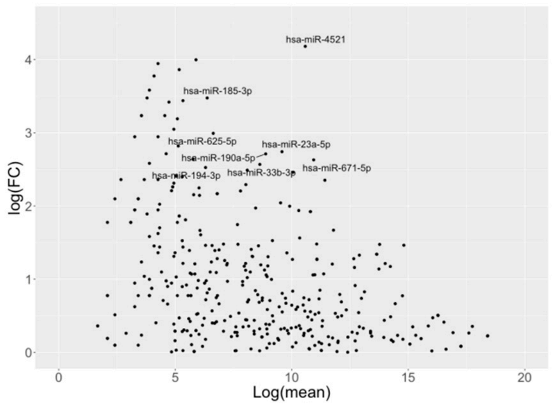

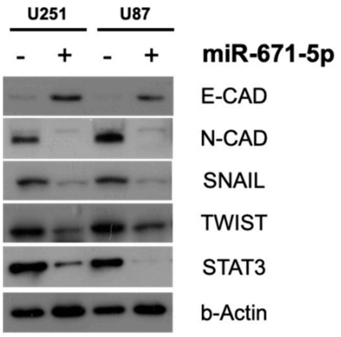

(**a**) (**b**)

Figure S1. (**a**) Small RNA sequencing of MSI1 knockdown GBM U87MG cell. (**b**) Western blotting for EMT-related proteins validation of mRNA.
